# Low-Power NIR-Triggered Photothermal Inactivation of *Pseudomonas aeruginosa* with Polypyrrole Nanoparticles

**DOI:** 10.3390/polym17111442

**Published:** 2025-05-23

**Authors:** Melina D. Gil, Silvestre Bongiovanni Abel, César A. Barbero, Natalia S. Paulucci, Edith I. Yslas

**Affiliations:** 1 Department of Molecular Biology, FCEFQyN-National University of Río Cuarto, Ruta Nacional Nº 36 Km 601, Río Cuarto 5800, Argentina; 2 Biomedical Polymers Division, Research Institute for Materials Science and Technology (INTEMA), National University of Mar del Plata (UNMdP)-National Scientific and Technical Research Council (CONICET), Av. Colón 10850, Mar del Plata 7600, Argentina; 3 Department of Chemical and Food Engineering, Faculty of Engineering, National University of Mar del Plata (UNMdP), Av. Juan B. Justo 4302, Mar del Plata 7600, Argentina; 4 Research Institute for Energy Technologies and Advanced Materials (IITEMA), National University of Río Cuarto (UNRC)-National Scientific and Technical Research Council (CONICET), Ruta Nacional Nº 36 Km 601, Río Cuarto 5800, Argentina; 5 Research Institute for Environmental Biotechnology and Health (INBIAS), National University of Río Cuarto (UNRC)-National Scientific and Technical Research Council (CONICET), Ruta Nacional Nº 36 Km 601, Río Cuarto 5800, Argentina

**Keywords:** polypyrrole nanoparticles, photothermal inactivation, antibacterial nanomaterials, antimicrobial resistance, *Pseudomonas aeruginosa*

## Abstract

Conducting polymer (CP) nanoparticles have emerged as innovative materials for biomedical applications, particularly due to their safe interaction with biological systems. This study focuses on the synthesis, morphological, and spectroscopic characterization of polypyrrole nanoparticles (PPy-NPs) as photoactivatable agents under near-infrared (NIR) radiation for the inactivation of pathogenic bacteria. We successfully synthesized uniform nanoparticles (~180 nm) with strong absorption in the NIR region. A comprehensive characterization was performed using electron microscopy, dynamic light scattering, X-ray diffraction, and UV–Vis and infrared spectroscopy. The microbiological evaluation focused on elucidating the inactivation mechanism of *Pseudomonas aeruginosa*, particularly through oxidative stress induction, metabolic activity alteration, and cell membrane disruption. Our results highlight the significant potential of PPy-NPs as photoactivatable agents for the targeted inactivation of pathogenic microorganisms, underscoring their promising applications in antimicrobial surface coatings.

## 1. Introduction

Nowadays, antimicrobial resistance (AMR) is considered an important issue that needs to be solved promptly [1,2,3]. Since the “golden era” of antibiotics, the widespread use of these compounds has led to the emergence and prevalence of antibiotic resistance [4,5]. Several microorganisms have developed different mechanisms to resist the action of antibiotics. For example, changes in membrane permeability, target modifications, or enzymatic inactivation are possible mechanisms to evade the action of these substances [6,7,8]. In particular, *Pseudomonas aeruginosa* (*P. aeruginosa*) is a typical Gram-negative opportunistic pathogen found in various environments, particularly in water, high-humidity areas, and different hospital settings and places [9]. This bacterial strain can cause serious infections in a wide range of cells and tissues due to its ability to adhere to surfaces and form biofilms [10,11]. The resistance of *P. aeruginosa* is primarily attributed to the production of β-lactamases and the modification of targets for antimicrobial compounds such as antibiotics [12,13,14].

Among various strategies to combat resistance, the development of antibacterial photothermal inactivation (a-PTI) has gained significant attention in recent years [15,16]. This approach type involves the application of radiation in the range of 700–1200 nm in wavelength and the subsequent absorption of this radiation by a photothermal agent. This agent converts the absorbed energy into heat through non-radiative mechanisms [13]. Several kinds of compounds and nanomaterials have been used in this sense. For instance, metal nanomaterials (Au nanorods, Ag nanoparticles, CuS, MoS_2_, etc.) have demonstrated their abilities to participate in a-PTI mainly due to the optical properties exhibited due to the surface plasmon resonance and a non-radiative relaxation of a semiconductor [16,17,18,19,20]. Furthermore, carbon-based nanomaterials, including carbon nanotubes (CNT), carbon dots (CD), graphene oxide (GO), and their derivatives and composites, were explored for similar purposes due to the strong absorption in the whole electromagnetic spectrum [18,21,22,23,24].

Conducting polymers (CP) are organic macromolecules that have also shown promising potential because of their versatility and possibility of being functionalized using several reaction types [25,26]. The most representative conducting polymers include polyaniline (PANI), polypyrrole (PPy), and polythiophene (PTh). Into this kind of materials, nanoparticles have an important advantage due to the high surface-to-volume ratio added to properties related to electromagnetic absorption (including microwaves, radiofrequency, and near-infrared radiation (NIR) and biocompatibility [27,28]. For example, the use of polyaniline nanoparticles (PANI-NPs) has been widely explored. In a previous report, our group developed nanometric spherical structures based on PANI with the potential to be used as a photothermal agent under NIR irradiation [13,29]. The a-PTI induced the *P. aeruginosa* death in more than 80%, demonstrating the cell death by DNA damage. On another side, other kinds of PANI-NPs were synthesized and functionalized to provide fluorescence added to the capability to inactivate the pathogen strain [30]. The use of similar PANI-NPs for incorporation into polymeric matrices (e.g., hydrogels) allowed researchers to also explore possibilities to develop wound dressings [31]. Finally, the loading of PANI-NPs systems allowed researchers to combine a-PTI with other treatments in drug delivery applications [32]. The advances mentioned are promising for exploration of theragnosis platforms.

Although methods for PPy functionalization are limited compared to other CPs such as PANI, the good electroactivity and conductivity at neutral pH becomes interesting in terms of their use in biomedical applications [33]. In this regard, the design of novel approaches is interesting because it allows us to overcome the insolubility of PPy in aqueous media. Moreover, PPy and its nanostructures exhibit several advantages over other conventional CPs. Notably, they present more accessible and straightforward synthetic pathways, particularly when compared to PT, as well as improved stability under aqueous and physiological conditions [34,35,36]. Additionally, PPy demonstrates favorable biocompatibility and minimal cytotoxicity, making it a suitable candidate for biomedical applications compared with PANI and its nanostructures [37,38,39]. With this aim, and considering the advantages of nanoparticles’ size and properties, in this study, we report a synthetic route to easily obtain PPy-NPs in a reproducible and controlled way and their use as antibacterial material under NIR exposition. Our final objective is demonstrating the synergistic combination of this nanomaterial type with a non-invasive radiation type for the biological tissues, studying particularly the interaction with the bacteria cell envelope to stablish a mechanism of pathogen death.

## 2. Materials and Methods

### 2.1. Chemicals

Pyrrole (Py, reagent grade, 98%) and ammonium peroxydisulfate (APS) were provided by Merck. Poly(vinylpyrrolidone) (PVP, MW = 360 kDa) was purchased from Fluka. Deionized water (Milli-Q quality, resistivity = 18 MΩ cm^−1^) was used to perform the experiments. Py was vacuum-distilled before use. All other chemicals were used without further purification as received from suppliers.

### 2.2. Bacterial Strains and Growth Conditions

The Pseudomonas aeruginosa PAO1 strain was used, preserved in glycerol at −80 °C. For subsequent assays, an aliquot was thawed and streaked onto solid LB medium using the exhaustion streaking technique, followed by incubation at 37 °C for 24 h. *P. aeruginosa* will be used to evaluate the antibacterial activity of nanomaterials. Luria–Bertani broth (LB broth) and Luria–Bertani agar (LB agar) will serve as nutrient sources for cultivation. *P. aeruginosa* will be inoculated in LB broth and incubated overnight at 37 °C in a sterilized shaking incubator (inoculum). The culture will then be adjusted to achieve bacterial growth in the exponential phase before use, with a final bacterial concentration of 1×10⁶ CFU mL^−1^.

### 2.3. Methods

#### 2.3.1. PPy-NPs Synthesis

PPy-NPs were synthesized by modifying a previously reported procedure [27]. Briefly, a homogenized solution containing the stabilizer agent (PVP 2% *w*/*v*) was prepared in water by stirring at 200 rpm for 30 min. Separately, 0.08 mol L^−1^ of APS was weighed. An amount of 0.043 mol L^−1^ of Py was added to the stabilizer solution, and stirring was continued for 10 min. Finally, the APS was added, and the polymerization process occurred. The obtained PPy-NPs dispersion was black color, and after 30 min, the process ended. The entire synthetic procedure was carried out at room temperature. PPy-NPs were then purified by 4 kDa cutoff for 72 h, changing the water twice per day. After purification, the nanoparticles were freeze-dried in a Virtis lyophilizer for 48 h.

#### 2.3.2. DLS and Z-Potential

Synthesized PPy-NPs were measured using a DLS device to determine the hydrodynamic diameter and polydispersity index (PDI). For this purpose, a Malvern Zetasizer Nano S90 ZEN1690 (Worcestershire, UK) device was used, measuring at a scattering angle of 90°. The surface charge of the PPy-NPs was evaluated using the same device in a polystyrene cell.

#### 2.3.3. Electronic Microscopy (SEM and TEM)

Electronic microscopy was used to observe the nanoparticles’ morphology. For Scanning Electronic Microscopy (SEM), an FE-SEM ZEISS Crossbeam 350 (Oberkochen, Germany) microscope was used. Observation at several magnifications was made after 1 min of gold sputtering. In the case of Transmission Electronic Microscopy (TEM), the examination was performed using a JEOL JEM 2100 (Tokio, Japan) instrument after depositing a diluted PPy-NPs dispersion on a copper grid.

#### 2.3.4. Spectroscopic Characterization (UV–Visible and FTIR)

Evaluation of PPy-NPs absorption was performed by measuring in a Hewlett-Packard 8453 diode array UV–Visible spectrophotometer (Waldbronn, Germany) using quartz cells (path length of 1 cm). The FTIR spectrum was measured in ATR mode placing the freeze-dried powder of PPy-NPs. The spectra were conducted in a Nicolet 7600, Thermo Scientific Inc. apparatus (Waltham, MA, USA) setting 64 scans with a resolution of 4 cm^−1^.

#### 2.3.5. X-Ray Diffraction (XRD)

XRD examination of PPy-NPs was performed using a Panalytical X’Pert PRO X-Ray Diffractometer (Worcestershire, UK) at 30 mA and 40 kV equipped with a Cu-Kα radiation source (λ ~ 1.54 Å). Scanning was conducted at 1° min^−1^, ranging from 5 to 60°.

#### 2.3.6. Thermogravimetric Analysis (TGA)

The weight loss of the nanoparticles under temperature increasing was evaluated using a TGA-50 Shimadzu (Kioto, Japan) thermogravimetric analyzer. The experiment was carried out from room temperature to 800 °C under nitrogen (N_2_) atmosphere at 10 °C min^−1^ heating rate.

#### 2.3.7. Evaluation of *P. aeruginosa* Viability at Different Concentrations of PPy-NPs

An overnight culture of *P. aeruginosa* was inoculated into 20 mL of liquid LB medium and then an aliquot was incubated in medium fresh and finally incubated shaking at 37 °C for 6 h to reach exponential phase. Subsequently, 100 μL of this culture was incubated with different PPy-NPs concentrations (0.102 mg mL^−1^, 0.255 mg mL^−1^, and 2.55 mg mL^−1^) and a saline solution (SF) control. Then, they were incubated at 37 °C for 24 h, to evaluate the viability using the droplet microdilution method. On the other hand, a *P. aeruginosa* culture was inoculated from a plate into 20 mL of liquid LB medium and incubated under agitation at 37 °C for 6 h until reaching the exponential growth phase. Subsequently, 100 μL of this culture was spread onto an LB agar plate using a Drigalski spatula. Wells were punched into the agar to add 20 μL of different concentrations of PPy-NPs (0.025 mg mL^−1^, 0.255 mg mL^−1^, and 2.55 mg mL^−1^) and a saline solution (SF) with a control. Finally, the plates were incubated at 37 °C for 24 h, after which inhibition halos were measured [40].

#### 2.3.8. Photothermal Irradiation Set-Up

The photothermal effect generated by NIR irradiation was evaluated by exposing a dispersion sample in a 96-well plate to an 850 nm LED with a power of 3 W. The temperature increase and decrease of the samples were monitored using a thermographic camera (Testo 868, Titisee-Neustadt, Germany) at 1-min intervals. Luria–Bertani (LB) medium was used as a blank to confirm that the photothermal effect was due to the irradiation of PPy-NPs.

#### 2.3.9. Antibacterial Photothermal Inactivation (a-PTI)

For the assays, *P. aeruginosa* is cultured at 37 °C under agitation until reaching the exponential phase. The in vitro experiments include four treatments: (1) control bacteria (control), (2) bacteria incubated with PPy-NPs and irradiated (a-PTI), (3) bacteria incubated with PPy-NPs in darkness (dark control), and (4) bacteria exposed only to irradiation (light control). The bacterial count (CFU mL^−1^) is determined 5 h post-irradiation using the droplet microdilution method [13].

On the other hand, to evaluate bacterial viability, *P. aeruginosa* from each treatment group (control, PPy-NPs + irradiation, PPy-NPs in darkness, and irradiation-only) is stained using the LIVE/DEAD BacLight Bacterial Viability Kit (Life Technologies, Carlsbad, CA, USA) according to the manufacturer’s protocol [41]. The stained samples are then visualized under a fluorescence microscope (Nikon Eclipse 50i, Tokio, Japan). Samples are prepared for fluorescence microscopy by mounting bacterial suspensions on slides with mounting medium and applying coverslips.

#### 2.3.10. Determination of Reactive Oxygen Species (ROS)

The ROS generation was measured according to the previously described method using DCFH-DA (Sigma-Aldrich, St. Louis, MO, USA). Bacterial cells (10^6^ CFU mL^−1^) were treated from each treatment group (control, PPy-NPs + irradiation, PPy-NPs in darkness, and irradiation-only) at the required temperature for 5 h post-irradiation. After incubation, cells were centrifuged for 5 min at 5000× *g*. A bacteria pellet was re-suspended in 30 μg mL^−1^ of dye solution in PBS and left in a shaking incubator at 37 °C for 45 min. Upon entry of DCFH-DA into cells, an oxidation reaction between the non-fluorescent dye and intracellular ROS occurred, and the dye turned into a highly fluorescent compound called 2,7′ dichlorofluorescein. Then, it was stained by 15 min with Hoescht. Cells were then centrifuged and washed two times to remove the DCFH outside the cell and re-suspended in 450 μL of fresh PBS. Finally, we proceeded to observe the cells under fluorescence microscope [42,43].

#### 2.3.11. Metabolic Activity Analysis by MTT Assay

Following a 5-h post-a-PTI incubation, a colorimetric assay was performed using MTT reagent (3-(4,5-dimethylthiazol-2-yl)-2,5-diphenyltetrazolium bromide), which undergoes redox reactions with compounds produced by dehydrogenase enzymes, reducing MTT to formazan (5-(4,5-dimethylthiazol-2-yl)-1,3-diphenylformazan). This formazan product exhibits an absorbance peak at 570 nm. Absorbance values are directly proportional to the metabolic activity of the microorganism. For the assay, the four evaluated conditions (control, dark control, light control, and a-PTI) were transferred to sterile microtubes. Then, 20 μL of MTT reagent was added to each tube, followed by incubation at 37 °C for 1.5 h in the dark. After incubation, the samples were centrifuged at 5000 rpm for 5 min, the supernatants were discarded, and the pellets were resuspended in 400 μL of DMSO. Finally, absorbance was measured at λ = 570 nm using a Beckman DU 640 (Brea, CA, USA) spectrophotometer [44].

#### 2.3.12. Determination of Fatty Acid by Gas Chromatography (GC)

Fatty acid methyl esters (FAMEs) were obtained according to Cesari et al. [45]. An aliquot of the lipid extract was treated with 1 mL of boron trifluoride (20% BF_3_ in methanol) for 120 min at 80 °C. Then, a water/hexane mixture (1:2, *v*/*v*) was added, vortexed, and the upper phase containing the FAMEs was collected. The analysis was performed using a 5890 II gas chromatograph (Hewlett-Packard, Palo Alto, CA, USA) equipped with a highly polar column, HP 88, of cyanopropyl (length, 60 m; inner diameter, 0.25 mm; film thickness, 0.2 µm), and a flame ionization detector. Gas chromatograph conditions were as follows: injector temperature, 250 °C; detector temperature, 300 °C; and nitrogen as carrier gas. The temperature was programmed at 120 °C for 1 min and then increased by 10 °C min^−1^ to 175 °C for 10 min, 5 °C min^−1^ to 210 °C for 5 min, and 5 °C min^−1^ to 230 °C for 5 min. The chromatograms obtained were analyzed and the fatty acids were identified by comparing retention times to commercial standards (Sigma Chemical Co., St. Louis, MO, USA). The area of each peak was used to determine relative quantities.

#### 2.3.13. Statistical Analysis

All analytical determinations were performed in triplicate and four experiments independently, and the results are expressed as mean ± standard error. Data were subjected to an analysis of variance (ANOVA) followed by a comparison of multiple treatment levels using Tukey’s post hoc test. The statistical significance was assessed by Origin 2024 software, at a significance threshold value of *p* < 0.05.

## 3. Results and Discussion

### 3.1. Synthesis and Characterization of PPy-NPs

**Figure 1:**
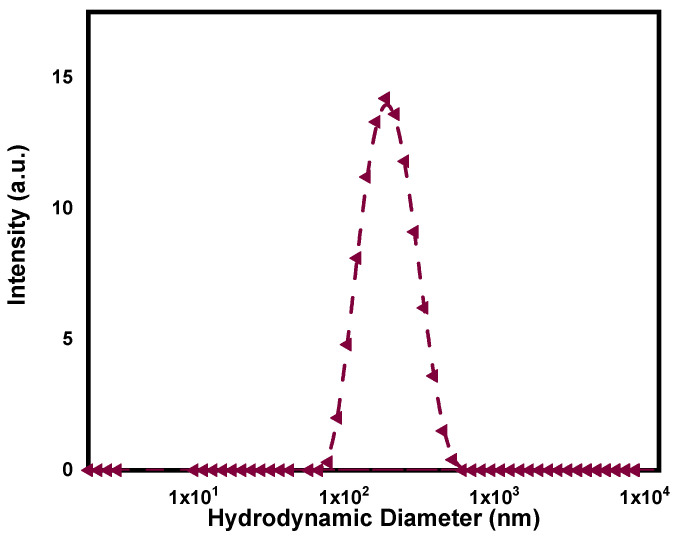
Dynamic light-scattering plot (as intensity) of synthesized PPy-NPs.

**Figure 2:**
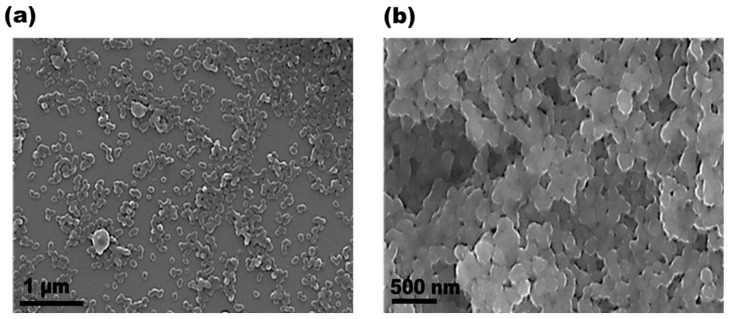
SEM micrographs of PPy-NPs at different magnifications: (**a**) scale bar: 1 µm; (**b**) scale bar: 500 nm.

**Figure 3:**
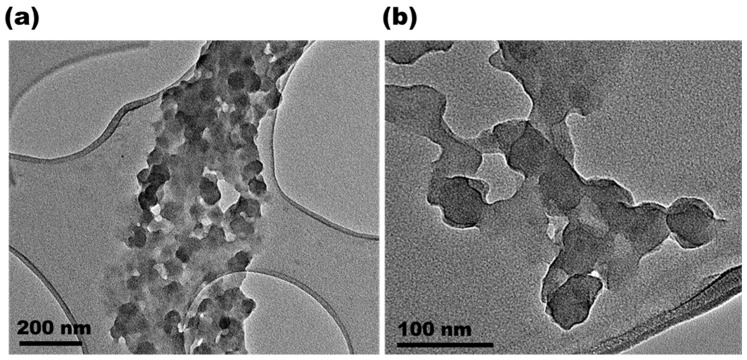
TEM micrographs of PPy-NPs at different magnifications: (**a**) scale bar: 200 nm; (**b**) scale bar: 100 nm.

After the synthesis, PPy-NPs were freeze-dried for conservation as a powder. Regarding size, the hydrodynamic diameter measured by DLS resulted in 183 ± 5 nm, displaying a monomodal distribution as shown in Figure 1. The PDI for the PPy-NPs dispersed in water was 0.188, indicating a good value for the proposed application and confirming that the sample was monodispersed in aqueous media. The surface charge of the synthesized particles resulted in a slightly negative value (−5.2 ± 0.6 mV). Despite the above-mentioned characterization, the nanoparticles were also observed by electronic microscopy techniques to investigate morphology. In this regard, the SEM images (Figure 2) and TEM images shown in Figure 3 confirmed the good monodispersity and regular shape of the PPy-NPs. It is noteworthy the difference of size comparing to the DLS result that is due to the solvation of the particles in a liquid environment and the possibility of aggregation of two or more particles.

**Figure 4:**
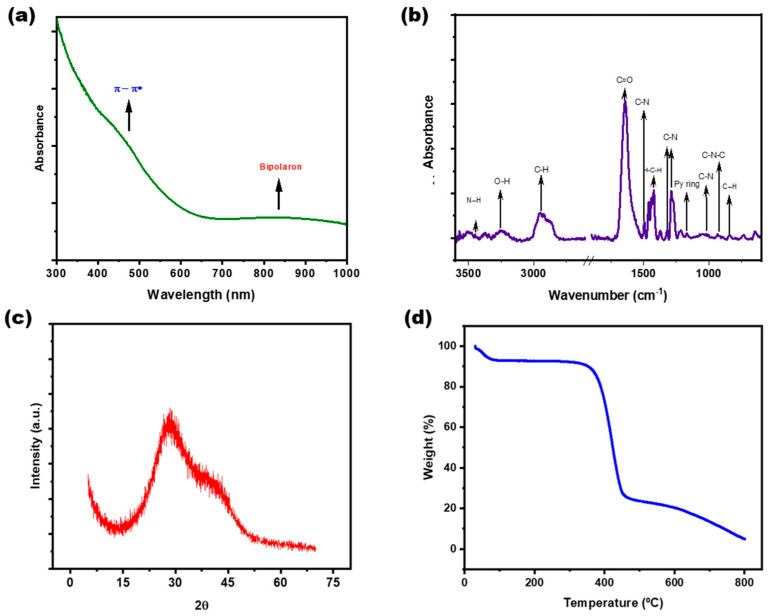
General characterization of the PPy-NPs. (**a**) UV–visible spectrum for nanoparticle dispersion in water, (**b**) FTIR spectrum of PPy-NPs powder, (**c**) XRD plot of freeze-dried nanoparticles, (**d**) thermal degradation of the synthesized nanomaterial.

The material’s absorption in the near-infrared range was confirmed by measuring the UV–visible spectrum of the PPy-NPs dispersed in water. Figure 4a shows the presence of the bipolaron band between 650 and 1000 nm. A band at 430 nm, corresponding to π → π* transitions, was also identified. The FTIR spectrum further confirms the presence of CP in the nanoparticle structure. Figure 4b displays the characteristic bands of PPy, detailed as follows: the bands at 1402 cm^−1^ and 1305 cm^−1^ correspond to C-N stretching and in-plane C-H vibrations, respectively. Moreover, C-N stretching is observed at 1470 cm^−1^, while the peak at 1460 cm^−1^ is attributed to C=C stretching vibrations. The N-H stretching band is visible around 3400 cm^−1^ [46,47]. The =C−H bending vibrations of PPy appear at 1040 cm^−1^, and the symmetric stretching of C-N-C is detected at 910 cm^−1^. Furthermore, the band at 1178 cm^−1^ is assigned to the vibration of the pyrrole ring, while the band at 922 cm^−1^ corresponds to bipolaron formation due to charge carriers, attributed to C=N^+^−C stretching vibrations [48]. This finding aligns with previous reports [34,35]. Additional bands indicate the presence of PVP as a shell in the particle structure, mainly the band at ca. 1650 cm^−^^1^ corresponding to C=O [49]. The XRD results for PPy-NPs, shown in Figure 4c, reveal the amorphous nature of the electroactive polymer, as evidenced by a broad amorphous peak at 2θ = 25°, attributed to stacking phenomena. As part of the general characterization, the thermal degradation of PPy-NPs was evaluated using thermogravimetric analysis (Figure 4d). The results indicate that the material exhibits a weight loss of approximately 10% at 100 °C, due to the removal of retained water from the hydrophilic particle structure upon heating. Beyond 350 °C, the degradation of C, H, and N moieties in PPy, as well as the structural decomposition of PVP, becomes evident. At approximately 450 °C, a total weight loss of around 80% is observed. Finally, less than 10% of carbon residue remains at higher temperatures in the thermogram. These findings suggest that the material exhibits sufficient thermal stability for the proposed application.

**Figure 5:**
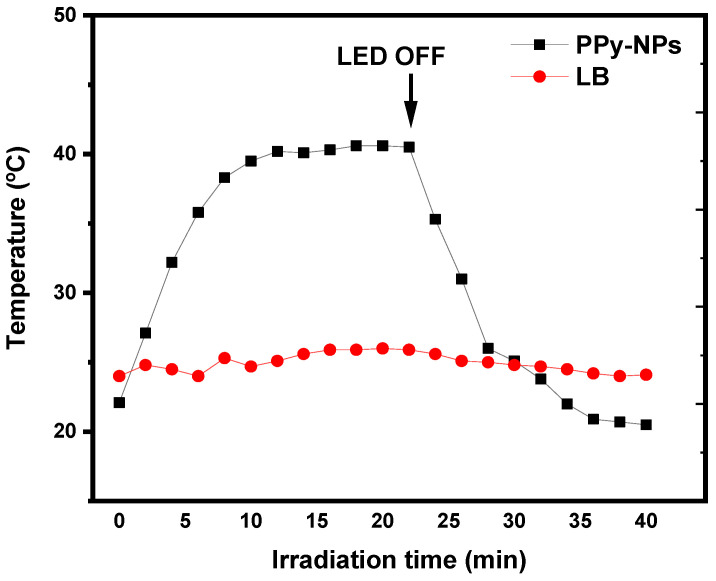
Photothermal heating effect on PPy-NPs under NIR for 20 min (LED ON) and 20 min (LED OFF) compared to the blank (LB medium). Arrow indicates the change of the NIR light (ON–OFF).

Under LED irradiation, the ability of PPy-NPs to generate a photothermal effect by converting electromagnetic radiation into heat was confirmed. A dispersion of PPy-NPs (2.55 mg mL^−1^) reached a ΔT° of 18.5 °C, as shown in Figure 5, demonstrating their photothermal capacity. After 20 min of irradiation, the LED was turned off, and the temperature returned to its initial value within less than 20 min. This result indicates that the heat generated is attributable to the photothermal effect produced by the PPy-NPs. For comparison, the temperature change for LB was only approximately 2 °C, confirming that the culture medium does not interfere with the photothermal process, a crucial consideration for biological assays of bacterial inactivation.

### 3.2. Antimicrobial Studies of PPy-NPs Against P. aeruginosa

#### 3.2.1. Effect of PPy-NPs Per Se on *P. aeruginosa* Viability

To determine the lethal effect of PPy-NPs dispersion on *P. aeruginosa*, two different assays were performed. The inhibition of bacterial growth by contact and diffusion of PPy-NPs in solid cell culture and viable cell counting was conducted after contact with the nanomaterial.

**Figure 6:**
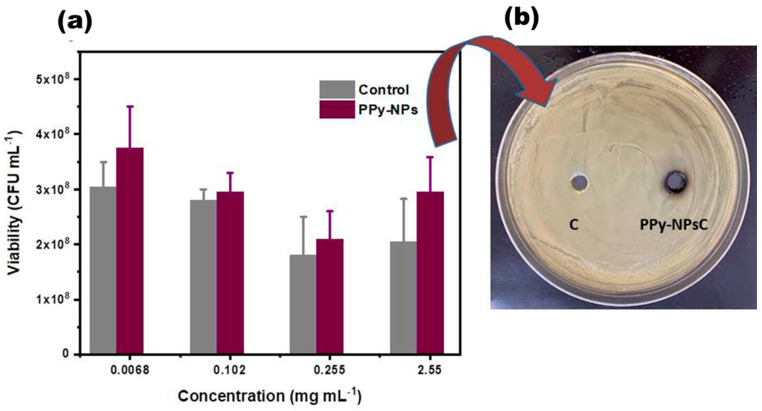
(**a**) Evaluation of *P. aeruginosa* viability at different concentrations of PPy-NPs by microdrop technique for counting in plate and incubation overnight at 37 °C and (**b**) evaluation of *P. aeruginosa* on inhibition halo of treatment control and bacteria incubated with PPy-NPs at 2.55 mg mL^−1^.

The results of the inhibition halo evaluation reveal that no inhibition zone was observed at the tested concentrations of PPy-NPs (0.025 mg mL^−1^, 0.255 mg mL^−1^, and 2.55 mg mL^−1^) using both methodologies. As shown in Figure 6b, a digital image confirmed the absence of the inhibition zone even at the highest concentrations (2.55 mg/mL). Additionally, Figure 6a demonstrates no significant variations in the viable bacteria counts across the different concentrations. These findings allow us to conclude that the conducting nanoparticles do not induce changes in the conditions required to inhibit *P. aeruginosa* growth, confirming that inhibition occurs exclusively through photothermal inactivation (see Section 3.2.2). This absence of antibacterial activity at low concentrations is consistent with previous reports. Almeida et al. determined the minimum inhibitory concentration (MIC) of PPy powder synthesized using different amounts of pyrrole, ammonium persulfate, and methyl orange [50]. The MIC for *E. coli*, comparing the two methodologies for obtaining PPy, showed the lowest concentration as 50 mg mL^−1^ assigned to the PPy II sample, while the MIC was about 100 mg mL^−1^ for PPy. Similarly, Kucekova et al. determined that for the MIC of colloidal PANI stabilized with PVP against *E. coli*, the MIC was 3500 mg mL^−1^ [51]. Considering that the concentrations evaluated in this study (≤2.55 mg mL^−1^) were substantially below the MIC values reported in the literature, our findings agree with prior research, confirming that PPy-NPs at low concentrations have an absence of intrinsic antibacterial activity.

#### 3.2.2. Bacterial Inactivation Assisted by NIR Irradiation on PPy-NPs

Photothermal inactivation of *P. aeruginosa* mediated by irradiated PPy-NPs was assessed at a concentration of 2.55 mg mL^−1^ (a concentration that does not exhibit lethal effects on its own). Viable cell counting was performed at 5 and 24 h post-irradiation.

**Figure 7:**
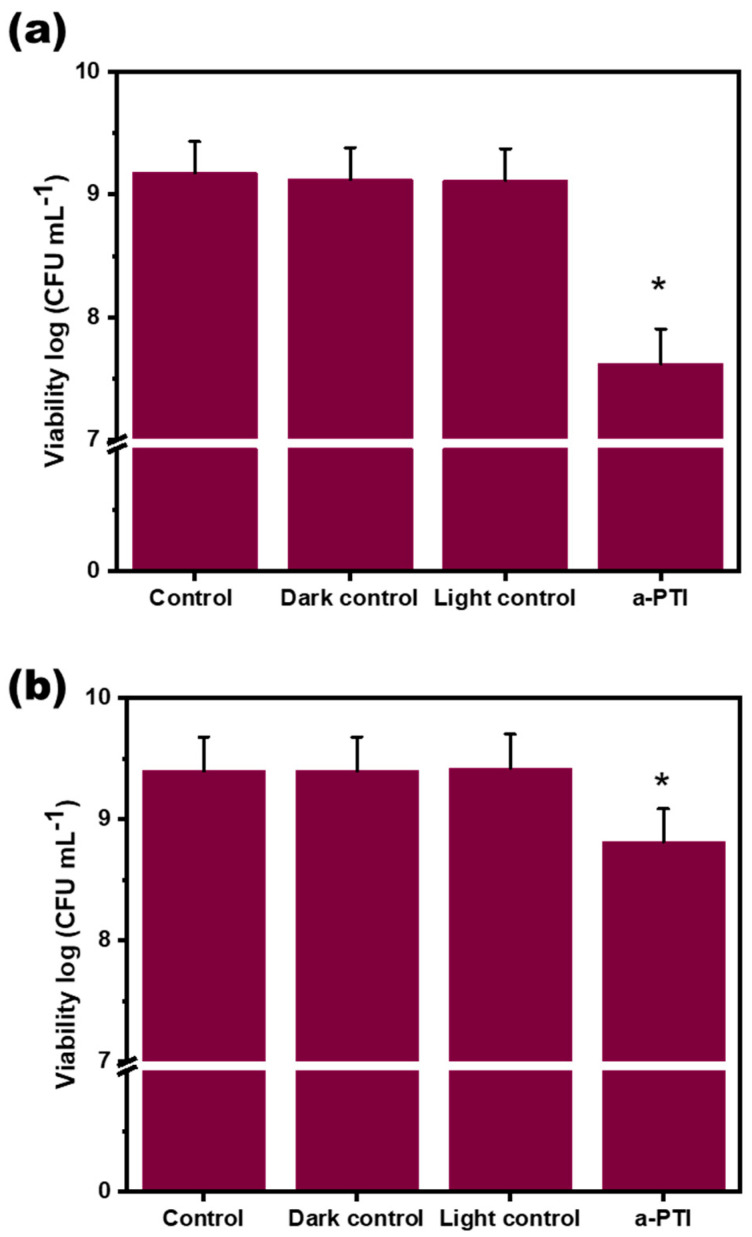
Antimicrobial effect of PPy-NPs on *P. aeruginosa* viability: (**a**) log(CFU mL^−1^) measured at 5 h after NIR exposure; (**b**) log(CFU mL^−1^) measured at 24 h after NIR exposure. Values represent mean ± SE. * indicates significant statistical differences between treatments. ANOVA analysis–Tukey Test (*p* < 0.005).

As shown in Figure 7, the bacterial counting results demonstrate that PPy-NPs alone do not induce changes in the viability of *P. aeruginosa* under control and dark control conditions at both 5 and 24 h. Moreover, irradiation with a low-power NIR LED for 20 min (light control condition) does not reduce bacterial viability in the absence of nanomaterials, yielding CFU mg mL^−1^ values similar to those observed under other controls previously mentioned. However, when PPy-NPs are combined with NIR light, bacterial viability decreased by approximately 1.55 log CFU mL^−1^ and 0.5 log CFU mL^−1^ at 5 and 24 h, respectively. These results suggest that photothermal inactivation occurs successfully. The observed differences in bacterial viability between the two time points can be explained due to a subset of bacteria entering a latent stage after treatment. Over time, these latent bacteria may recover their metabolic activity and resume cell division. This is evidenced by the 24-h post-irradiation viability, which is higher (in more than 1 log CFU mL^−1^) than the viability observed at 5 h after a-PTI treatment. Chen et al. reported that polypyrrole nanoparticles (PPy-NPs) exhibit strong absorption in the near-infrared (NIR) region, demonstrating notable photothermal conversion [52]. Their research established these nanomaterials as efficient photothermal agents for in vivo cancer therapy when activated by NIR irradiation. On the other hand, Behzadpour et al. observed bactericidal effects of a nanocomposite of C-dots and PPy (C-PPy nanocomposites) which showed that following C-PPy attachment to the bacteria surface, irradiation of near-infrared light resulted in a significant decrement in the bacterial cell viability due to photothermal lysis [53].

**Figure 8:**
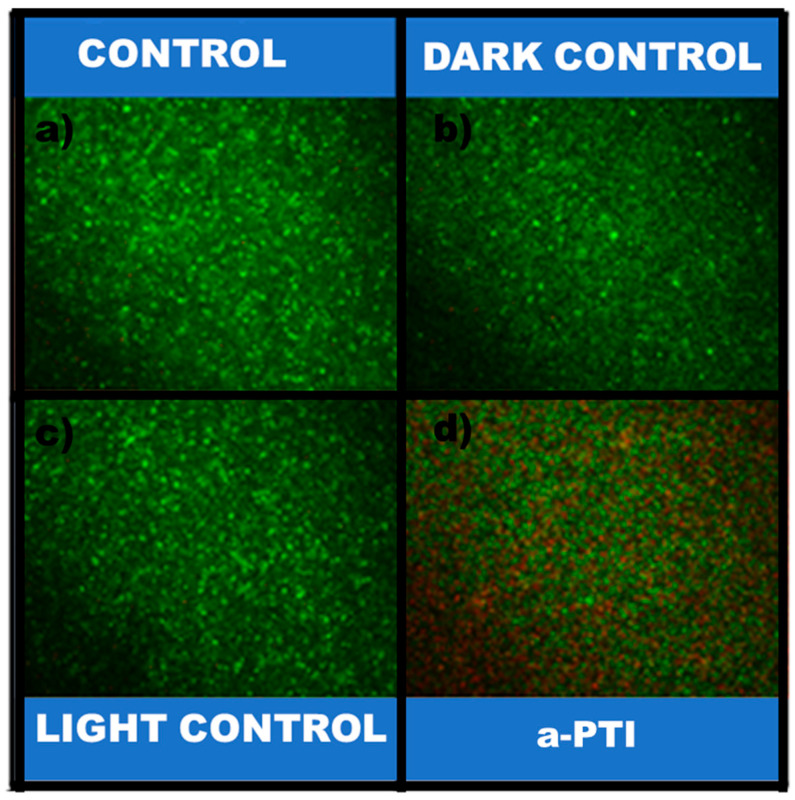
*P. aeruginosa* viability and cell membrane integrity: (**a**) *P. aeruginosa*; (**b**) *P. aeruginosa* + 20 min NIR; (**c**) *P. aeruginosa* + PPy-NPs; (**d**) *P. aeruginosa* + PPy-NPs + 20 min NIR. Scale bar: 50 µm. Samples were stained and evaluated after staining them with LIVE/DEAD Bac Light^®^ Bacterial Viability Kit (Thermo Fisher, Waltham, MA, USA). Live bacteria appear as green, dead bacteria as red.

The membrane integrity test of *P. aeruginosa* was assessed using fluorescence microscopy. As shown in Figure 8, micrographs of control groups (dark and light condition, as well as bacteria-only controls) reveal predominantly viable cells. In contrast, NIR irradiation in the presence of PPy-NPs induce significant bacterial death, evidenced by membrane integrity loss. Notably, neither PPy-NPs alone nor NIR irradiation alone produced this bactericidal effect, confirming that the synergistic combination of both NIR irradiation and PPy-NPs is required. These results align with the viable cell counts and metabolic activity data previously presented.

**Figure 9:**
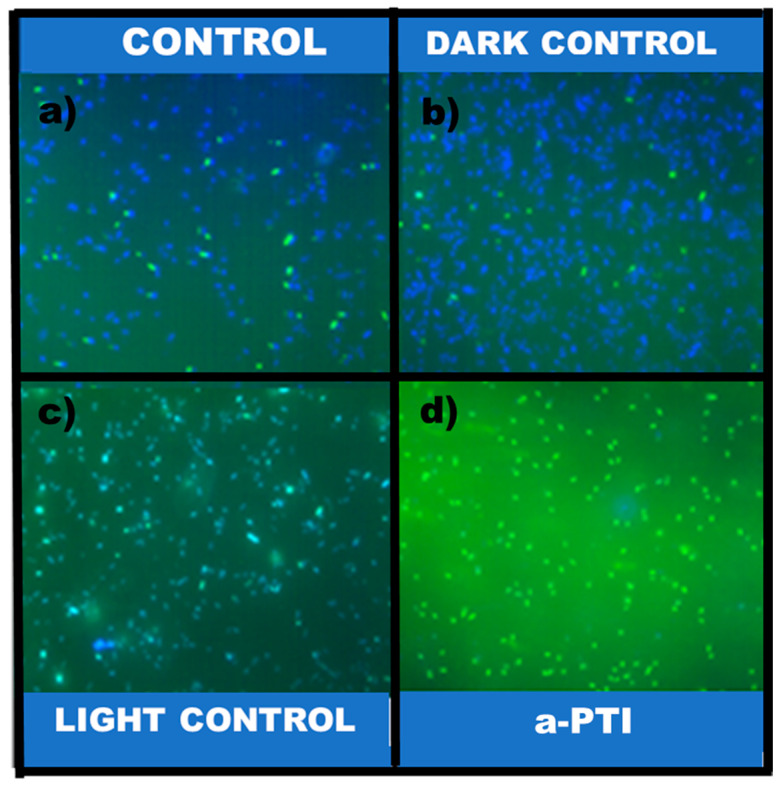
Effect of PPy-NPs irradiated with NIR in ROS production. (**a**) *P. aeruginosa*; (**b**) *P. aeruginosa* + 20 min NIR; (**c**) *P. aeruginosa* + PPy-NPs; (**d**) *P. aeruginosa* + PPy-NPs + 20 min NIR. Scale bar: 50 µm.

Further supporting these findings, reactive oxygen species (ROS) generation was assessed under the four experimental conditions (Figure 9). ROS production was detected exclusively in samples treated with PPy-NPs + NIR light, implicating oxidative stress as a key contributor to cell death. Control groups showed negligible ROS levels, reinforcing that the antibacterial mechanism depends on photothermal activation. These results are consistent with recent studies on conductive polymer-based nanomaterials. For example, Behzadpour et al. demonstrated that C-PPy induces a significant decrement in the bacterial cell viability of the pathogenic Gram-negative bacterium *P. aeruginosa* due to photothermal lysis, accompanied by increased protein leakage and intracellular ROS levels. Our study reveals that the photothermal effect predominates at lower concentrations (≤2.55 mg mL^−^^1^), with ROS-mediated activity detected. Notably, our control experiments with non-irradiated PPy-NPs showed no significant antibacterial activity, supporting the photothermally activated nature of the bactericidal effect rather than intrinsic material toxicity. These results show the potential of PPy-NPs as therapeutic applications for photothermal inactivation, implying that bactericidal efficiency can be tuned by modulating NP concentration and irradiation parameters. Importantly, the absence of toxicity in non-irradiated PPy-NPs controls confirms that the observed effects are photothermally activated rather than intrinsic to the material.

**Figure 10:**
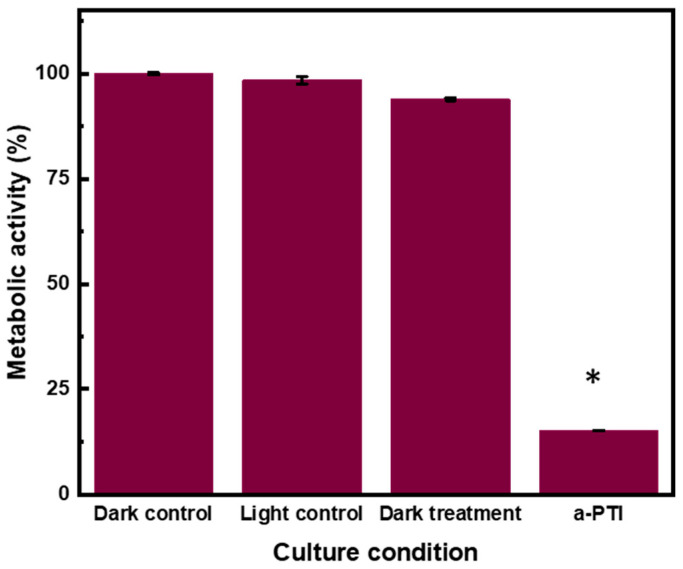
Percentage of metabolic activity of PPy-NPs under NIR irradiation measured by MTT against *P. aeruginosa*: *P. aeruginosa*, *P. aeruginosa* + NIR 20min, *P. aeruginosa* + PPy-NPs, and *P. aeruginosa* + PPy-NPs + NIR 20 min incubated for 5 h post-NIR at 37 °C. Values represent mean ± SE. * indicates significant statistical differences between treatments. ANOVA analysis–Tukey Test (*p* < 0.005).

To correlate the effects of NIR irradiation on *P. aeruginosa* (at a concentration of 2.55 mg mL^−1^) with their metabolic activity, the assay was performed a few hours post-irradiation, based on the results shown in Figure 10. The plot displays the percentages of metabolic activity, with similar values for conditions in absence of a-PTI, but an 80% reduction in NIR-treated bacteria exposed to PPy-NPs. These results indicate that the activity of dehydrogenase enzymes involved in the energy metabolic pathways remained unchanged in the absence of the nanomaterial combined with low-power light. However, after a-PTI treatment, the metabolic reactions essential for the growth of *P. aeruginosa* were significantly impaired when evaluated 5 h post-irradiation.

The cytoplasmic membrane serves as a crucial barrier, delineating the internal environment of the cell from the external surroundings [54]. Given its fundamental role in cellular integrity and function, it is a primary target for various external factors that influence cell viability. Bacteria have developed adaptive mechanisms to survive under adverse conditions by altering the composition of their membrane lipids and fatty acids. These modifications help maintain membrane fluidity, permeability, and functionality in response to environmental stresses such as temperature fluctuations, osmotic pressure, and antimicrobial agents. One key adaptive strategy involves modifying the degree of fatty acid unsaturation. For instance, an increase in unsaturated fatty acids enhances membrane fluidity, while a higher proportion of saturated fatty acids strengthens membrane rigidity under stress conditions [55]. Additionally, bacteria may incorporate cyclopropane fatty acids or alter the ratio of branched-chain fatty acids to further modulate membrane properties [56].

By analyzing the fatty acid content using chromatographic techniques, we were able to determine the effects of NIR radiation and subsequent photothermal inactivation on the cell envelope composition of *P. aeruginosa*. The evaluated strain exhibited a lipid profile predominantly composed of 16:0 (approximately 38%), 16:1 ^Δ9^ (approximately 32%), and 18:1 ^Δ11^ (approximately 25%). Additionally, the fatty acid 18:0 was present in smaller amounts, constituting about 5% of the total. These findings are consistent with other reports in the literature. Mezo et al. studied in detail the fatty acid composition of *P. aeruginosa* PAO1 at 37 °C similar to the growth temperature used in this study. Similar to our results, the main fatty acids were saturated 16:0 and to a lesser extent 18:0 and monounsaturated 16:1 and 18:1 [57].

Our results indicate that contact between *P. aeruginosa* and PPy-NPs affects the fatty acid profile, leading to a 5% decrease in 16:1 ^Δ9^ and a 5.5% increase in 18:0 under dark conditions. In light exposure condition, this effect was more pronounced due to the impact of NIR radiation, resulting in an approximately 16% decrease in 16:1 ^Δ9^ and a 22.5% increase in 18:0. Additionally, reductions of 6.3% and 7.2% were observed in 16:0 and 18:1 ^Δ11^, respectively. All results are summarized in Table 1. In summary, we demonstrate that exposure of this strain to PPy-NPs induces changes in the fatty acid composition; however, this effect was more pronounced when the bacteria were exposed to NIR light. The U/S ratio decreases progressively (1.33 → 1.05 → 0.7). This suggests a shift towards a more rigid membrane, reducing fluidity under PPy-NPs + NIR radiation conditions. The biological interpretation of these results is not straightforward and could be related to several aspects resulting from the joint application of PPy-NPs + NIR radiation. For example, the increase in temperature that occurs during the process could be responsible for adaptive changes at the level of fatty acids. It is well documented that the universal effect of temperature elevation causes in bacterial cell membranes an increase in the proportion of saturated fatty acids as observed in *P. aeruginosa* exposed to PPy-NPs+, to counteract the damaging effects on membrane fluidity. On the other hand, the fact that the absence of radiation causes only minor changes in fatty acid composition also supports this hypothesis.

On the other hand, rather than an adaptive mechanism, the modifications observed could be an effect of the mechanism of action of this procedure itself, due to the generation of ROS. ROS can damage bacterial cell membranes by oxidizing unsaturated fatty acids, which can lead to cell death. However, the effect of ROS on bacteria depends on the fatty acid composition of the bacterial membrane. Compared to saturated fatty acids, the unsaturated fatty acids are vulnerable to oxidation. Unsaturated lipids are mainly located in the cell membrane, and the peroxidation of these molecules could therefore change the composition of the cell surface and contribute to bacterial death The dramatic drop in the U/S ratio suggests reduced bacterial viability, potentially enhancing the antimicrobial effect of PPy-NPs + NIR treatment. Similar results were observed in *S. aureus* exposed to blue irradiation, which induces high ROS production, with a decrease in unsaturated fatty acids, demonstrating the relationship between ROS production and modification of the bacterial membrane fatty acid profile [58].

The *U/S ratio* (unsaturated/saturated ratio) refers to the proportion of unsaturated (U) to saturated (S) fatty acids in a biological membrane.

**Scheme 1:**
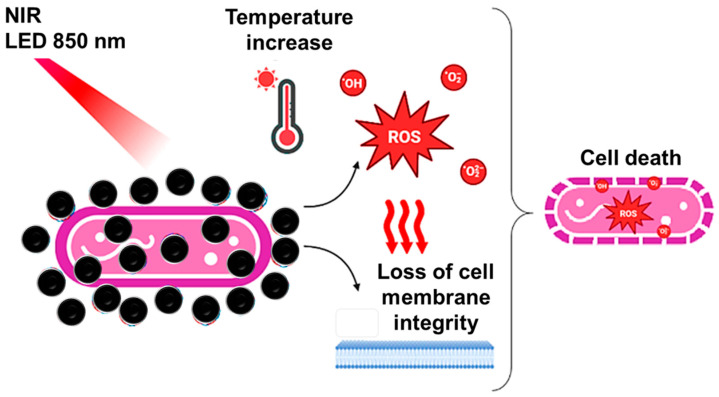
Summary of the mechanism proposed in this research for the antibacterial photothermal effect of PPy-NPs against *P. aeruginosa*.

The findings of our study allow us to propose a mechanism for the a-PTI of the synergistic combination of PPy-NPs and low-power NIR light against *P. aeruginosa* as is shown in Scheme 1. In this sense, the radiation is capable of producing the heating of the conductive nanogent with the subsequent generation of bacterial death produced by the ROS generation and the loss of metabolic activity of the microorganism with modification of the fatty acid profile.

## 4. Conclusions

Herein, we reported a simple synthesis of water-dispersible PPy-NPs with appropriate size, uniformity, and low polydispersity. The physicochemical characterization of the obtained nanoparticles revealed the absorption in the NIR range of the electromagnetic spectra and the thermal stability at operational temperature. We demonstrated the ability of the PPy-NPs as photothermal agent, allowing the increase of more than 17 °C in only 20 min. The low power of the NIR source is highlighted, which is important from the point of view of practical applications. Regarding the antimicrobial inactivation, the performance of the PPy-NPs (nontoxic per se at the tested concentrations for the bacterial strain) resulted as efficient for the inhibition of *P. aeruginosa*, decreasing its viability and its metabolic activity. The photothermal process induces the bacterial death by ROS generation and cell membrane integrity loss, also modifying the fatty acids profile of the membrane. As a perspective, we believe it is possible to incorporate the herein synthesized particles in different polymeric matrices types (e.g., hydrogels, polymeric films, electrospun mats, etc.) composed by hydrophilic polymers or blends with the aim to serve as a support, helping the fight against pathogenic-resistant bacteria and antibiotic resistance mechanisms.

## Figures and Tables

**Table 1:** Fatty acid composition of *P. aeruginosa* at the different conditions. Values represent the mean of three independent determinations ± S.E.M. * indicates statistically significant differences with respect to the control (*p* < 0.05).

Fatty Acid	*P. aeruginosa* (Control)	*P. aeruginosa* + PPy-NPs (Dark Control)	*P. aeruginosa* + PPy-NPs + NIR (a-PTI)
16.0	37.95 ± 1.08	38.27 ± 0.33	31.57 ± 1.80 *
16.1 ^Δ9^	31.59 ± 1.47	26.55 ± 0.79 *	15.70 ± 1.15 *
18.0	5.06 ± 2.49	10.49 ± 1.59 *	27.53 ± 3.10 *
18.1 ^Δ11^	25.38 ± 0.07	24.69 ± 1.13	18.18 ± 0.95 *
U/S	1.33	1.05	0.70

## Data Availability

The datasets generated and/or analyzed during the current study are available from the corresponding author upon reasonable request.
